# The impact of social exclusion on malevolent creativity in college students: the mediating role of aggression and the moderating effect of team sports participation—evidence from universities in Shanghai

**DOI:** 10.3389/fpsyg.2026.1757500

**Published:** 2026-02-20

**Authors:** Jiahui Shi, Hui Qiu, Jingjing Li, Bing Liu

**Affiliations:** 1 Physical Education College, Shanghai University, Shanghai, China; 2 Physical Education Research Center, Shanghai University, Shanghai, China; 3 School of Physical Education, Shanghai University of Traditional Chinese Medicine, Shanghai, China

**Keywords:** aggression, college students, malevolent creativity, social exclusion, team sports participation

## Abstract

**Introduction:**

This study examined how social exclusion relates to malevolent creativity among college students and tested the mediating role of aggression and the moderating role of team sports participation.

**Methods:**

A total of 1,288 students (656 males, 632 females) from universities in Shanghai completed validated scales assessing social exclusion, aggression, malevolent creativity, and team sports participation. Data were analyzed using the PROCESS macro with bootstrapping, controlling for gender, grade, and age.

**Results:**

Social exclusion significantly and positively predicted malevolent creativity, and that aggression partially mediated this association. Social exclusion remained a significant predictor of malevolent creativity after including aggression, while the indirect effect via aggression was also significant, indicating a partial mediation pattern. Furthermore, team sports participation significantly moderated both the social exclusion–aggression and the aggression–malevolent creativity paths: the positive associations were stronger at low levels and weaker at high levels of team sports participation.

**Conclusion:**

These findings suggest that social exclusion may impact malevolent creativity in emerging adults partly by increasing aggression, whereas sustained engagement in team sports functions as a protective factor that mitigates this risk. The study highlights the importance of integrating sport-based interventions into campus mental health and behavioral prevention programs to guide creativity toward prosocial ends.

## 1 Introduction

Creativity is not inherently positive; its “dark side” also warrants close attention (Ning and Jing, 2016). Malevolent creativity refers to creative behavior carried out with the intention to harm or destroy, implemented through novel and effective means (Cropley et al., 2008). Similar to general creativity, malevolent creativity retains core features of novelty and originality, but it is essentially distinguished by its malevolent motivation and harmful consequences (Pearson, 2021). In recent years, the harms associated with malevolent creativity have shown a tendency toward emerging at younger ages and generalizing across multiple domains (Yang et al., 2025). Among college students, malevolent creative behaviors have increasingly permeated everyday life, becoming more diverse and covert in their manifestations (Xu et al., 2024). Survey data indicate that approximately 20.3% of adolescents have experienced school violence enacted through such creative means (Wei et al., 2019), including fabricating rumors via AI-based face-swapping technologies, exploiting code vulnerabilities for cyber extortion, and designing novel strategies of relational bullying. These behaviors not only pose direct threats to victims’ physical and mental health, but—because of their unpredictability and technology-enabled nature—also constitute profound challenges to campus safety and social stability.

For example, between 2021 and 2023, several computer science majors at a certain university fabricated an “online game development project,” forged game interfaces, and concocted profit data to defraud their classmates of several million RMB. This incident highlights the risk that college students may draw on their professional expertise and cognitive flexibility to redirect innovative potential toward malevolent goals. As college students are in a critical period of socialization and creative development (Shi et al., 2020), they tend to think actively and are willing to break conventions (Rhodes, 1961), yet their cognitive maturity and moral judgment are not fully developed. Consequently, they are more susceptible to environmental pressures and individual dispositions that may channel creativity toward harmful ends (Harris and Reiter-Palmon, 2015). Therefore, examining the antecedents of malevolent creativity among college students and exploring effective intervention strategies is of substantial practical significance for curbing harmful behaviors and fostering the prosocial development of creativity.

Building on the 6P model of malevolent creativity proposed by Cropley et al. (2010), the generative mechanism of malevolent creativity involves multidimensional interactions among Process, Product, Press, Person Properties, Person Motivation and Person Feelings among other factors. Among these dimensions, environmental factors may exert an even stronger influence on individual creativity than personal traits (Kandler et al., 2016). College students are in a critical period of socialization, during which their social and psychological functioning gradually matures and their need for interpersonal connectedness is particularly salient. Social exclusion, as a typical environmental stressor, undermines individuals’ sense of belonging and self-worth (Li and Qu, 2025), which may trigger psychological distress and hostile attributions. In turn, excluded individuals may engage in aggressive behavior as a means of defense or retaliation, ultimately catalyzing the emergence of malevolent creativity.

Moreover, social information processing theory suggests that situational cues shape the moral valence of creativity (Gutworth et al., 2018). Although existing research has shown that contextual factors such as competitive environments (Cropley, 2010) and unfair climates (Zhang et al., 2024) can significantly enhance malevolent creativity, it remains insufficiently understood whether positive environmental interventions can attenuate these negative effects by reshaping cooperative cognitions and perceptions of social support.

In recent years, scholars have increasingly focused on the moderating role of sport participation in college students’ psychological development. Participation in group sports, as a prototypical high–social interaction activity, can strengthen social connectedness and sense of belonging through shared goals, complementary roles, and cooperative experiences (Peng et al., 2025), and can buffer aggressive responses elicited by social exclusion (Schrader et al., 2022). The formation of sport-based friendships has also been shown to improve emotion regulation and behavioral control (Ma et al., 2023), thereby inhibiting the externalization of malevolent creativity. Therefore, the present study aims to examine the impact of social exclusion on malevolent creativity among college students and to provide theoretical and practical guidance for universities in China and beyond on how to use sport-based interventions to curb aggressive tendencies and channel students’ creative potential toward prosocial ends.

## 2 Literature review

### 2.1 Social exclusion and malevolent creativity among college students

Social exclusion refers to the phenomenon and process in which an individual is rejected, ignored, or ostracized by others or by a group in social interactions, resulting in unmet needs for belonging and relatedness (Du and Xia, 2008; MacDonald and Leary, 2005; Twenge et al., 2001). According to self-determination theory and the need-to-belong framework, humans possess a basic motivation to establish and maintain close relationships (Ryan and Deci, 2002). When this need for social connectedness is thwarted, individuals are likely to exhibit a range of negative emotional and behavioral reactions (Baumeister and Leary, 1995). As a common negative life event, social exclusion not only threatens one’s sense of security and social status, but also reflects injustices in the distribution of resources and rights, thereby constituting a typical unfair situation and stressor (Gabbiadini and Riva, 2018; Nezlek et al., 2015; Williams, 2007). Within the university campus as a key socialization context, students who experience marginalization or restricted participation and fail to obtain basic interpersonal connections are particularly susceptible to the adverse consequences of social exclusion (Li et al., 2024), with profound implications for their mental health and behavioral motivation.

Drawing on social information processing theory, individuals’ cognitions and behaviors are strongly shaped by the social context in which they are embedded (Zalesny and Ford, 1990). A substantial body of research has demonstrated that competitive or unfair environments significantly increase negative emotional experiences—such as anger, anxiety, depression, and social pain (Spencer and Rupp, 2009; Stillman et al., 2009; Wang et al., 2020)—and give rise to externalizing tendencies and aggressive responses (Shao and Zhang, 2021). Consequently, social exclusion makes individuals more likely to interpret social cues through a hostile lens and to generate creative ideas imbued with destructive or retaliatory intent (Baas et al., 2019). Using the Cyberball paradigm with a college sample, Wu (2021) found that excluded participants exhibited significantly higher levels of malevolent creative performance (including fluency, flexibility, and originality) in malevolent scenarios compared with the control group, thereby providing empirical support for the facilitating effect of social exclusion on malevolent creativity. Although existing studies have relatively consistently revealed a positive association between social exclusion and malevolent creativity, the underlying psychological mechanisms remain contested and insufficiently explored (Wu, 2021). Based on the General Aggression Model, the influence of social exclusion involves multiple layers—cognitive, affective, and physiological arousal—suggesting that its impact on malevolent creativity is likely to be a multidimensional and complex systemic process (Kapoor and Kaufman, 2022; Shao and Zhang, 2021).

In sum, social exclusion, as a typical environmental stressor, elicits negative emotional experiences and hostile cognitive processing, thereby increasing the likelihood that individuals express their creativity in malevolent ways and elevating their level of malevolent creativity. Accordingly, this study proposes the following hypothesis:

*Hypothesis 1*: Social exclusion positively predicts malevolent creativity among college students.

### 2.2 The mediating role of aggression

Aggression refers to behavioral manifestations or psychological tendencies whereby an individual intentionally harms another person or object, and can take multiple forms, including physical, verbal, symbolic, or written aggression (Fauzi et al., 2023; Zhu et al., 2019). Warren et al. (2011) further argue that aggression encompasses not only direct physical or verbal attacks, but also indirect interpersonal and social aggression. Its emergence is shaped by both internal emotional and cognitive processes and external environmental triggers (Warren et al., 2011).

Among various social contexts, social exclusion has been widely identified as a key external factor eliciting aggressive responses. Williams (2007) found that social exclusion undermines individuals’ sense of belonging and social connectedness, threatens their basic psychological needs, and thereby provokes negative emotional reactions such as anger and hostility (Williams, 2007). From the perspective of the General Aggression Model, social exclusion, as an external situational input, promotes aggressive behavior outputs by altering individuals’ internal states (Anderson and Bushman, 2002). In particular, when individuals perceive themselves as devalued or threatened, aggression may become a strategic coping response aimed at restoring a sense of control and social status (Hu et al., 2025; Yang et al., 2023). Empirical evidence shows that social exclusion increases hostile attributions toward others’ behaviors (Chester and DeWall, 2017) and significantly elevates impulsivity and aggressive tendencies (Hames et al., 2018). Thus, when individuals feel socially excluded and enter an angry or threatened emotional state, they are more likely to resort to aggression as a coping strategy.

Aggression, in turn, is considered a critical antecedent of malevolent creativity (Baas et al., 2019; Harris and Reiter-Palmon, 2015). Experimental work by Perchtold-Stefan et al. (2021) demonstrated that individuals with higher levels of aggression are more inclined to generate innovative ideas with retaliatory or manipulative intent in open-ended tasks. According to Trait Activation Theory, aggression as a personality trait is more readily activated in threatening situations and subsequently gives rise to goal-directed harmful behavior (Tett and Burnett, 2003). The General Aggression Model likewise posits that, under an aggressive state, individuals’ thinking becomes more negative and hostile (Anderson and Bushman, 2002), and aggression-related knowledge structures stored in memory are activated. These activated schemas further guide individuals to deploy creativity in the service of hostile or harmful goals, thereby fostering malevolent creativity (Li et al., 2022).

In summary, aggression is a psychological and behavioral response that is easily elicited by social exclusion, closely linked to hostile attribution and negative emotions, and serves as an important driving force of malevolent creativity. On this basis, the present study proposes the following hypothesis:

*Hypothesis 2*: Aggression mediates the relationship between social exclusion and malevolent creativity among college students.

### 2.3 The moderating role of team sports participation

Team sports are physical activities in which multiple participants pursue a shared goal through clearly defined role allocations and continuous interactive cooperation (Eime et al., 2013; Li and Qu, 2025). In this study, team sports participation refers to students’ regular engagement in organized team-based sports activities. According to self-determination theory (Ryan and Deci, 2000), participation in such activities can satisfy individuals’ need for relatedness and strengthen intrinsic motivation. Consistent with the need-to-belong framework (Baumeister and Leary, 1995), cooperative group experiences also reinforce individuals’ sense of belonging and expand their social support networks. A growing body of research indicates that sport participation provides college students with opportunities for social interaction (Ji et al., 2024), stress relief, and confidence building (Steptoe and Butler, 1996), and is closely associated with a reduced risk of mental health problems (Gore et al., 2001). In particular, team sports, as Scarapicchia et al. (2013) noted, offer a structured environment in which college students can build social support networks (Scarapicchia et al., 2013), share goals, cope with difficulties, and develop a strong sense of belonging (Opstoel et al., 2020). On this basis, the present study posits that team sports participation, as a positive environmental resource, may enhance social support and emotion regulation, thereby attenuating the impact of social exclusion on aggression and, in turn, suppressing the externalization of malevolent creativity.

More specifically, the social interactions and structured support system afforded by team sports can substantially mitigate the negative consequences of social exclusion. In a cross-sectional study, Boone and Leadbeater (2006) found that team sports participation was positively associated with perceived social acceptance and negatively associated with psychological distress (Boone and Leadbeater, 2006), suggesting that team sports help buffer the adverse effects of social exclusion on mental health by providing positive peer support and opportunities for skill development (Doré et al., 2020; Wang et al., 2025). Longitudinal evidence from Sabiston et al. (2016) further showed that adolescents who consistently participated in team sports exhibited lower levels of aggression in early adulthood, an effect that was not observed among those engaged primarily in individual sports (Sabiston et al., 2016). This pattern may be attributable to the way in which shared goals and role differentiation in sport strengthen individuals’ sense of belonging and reduce hostile attribution (Xu et al., 2025). Existing studies have also demonstrated that participation in team sports can enhance self-control and empathy through rule-based constraints and team cooperation (Colakoglu and Solak, 2014; Shachar et al., 2016). In cooperative sport settings, positive interactions among teammates effectively reduce the accumulation of anger and frustration (Pels and Kleinert, 2016), while structured supervisory environments such as school physical education classes lower the likelihood of aggressive behavior (Trajković et al., 2020a). Ramirez-Granizo et al. (2020) further reported that individuals with higher levels of team sports participation showed significantly lower levels of negative affect following rejection experiences and displayed stronger adaptability in self-control and problem-focused coping (Ramirez-Granizo et al., 2020). Taken together, these findings suggest that team sports participation may moderate the relationship between social exclusion and aggression. Accordingly, the present study proposes:

*Hypothesis 3a*: Team sports participation moderates the relationship between social exclusion and aggression among college students, such that the positive association between social exclusion and aggression is weaker at higher levels of team sports participation.

In addition, although there is currently no direct empirical evidence regarding the moderating role of team sports participation in the link between aggression and malevolent creativity among college students, related findings provide important clues. Trajković et al. (2020a,b), for example, found that although volleyball players and fitness enthusiasts reported similar levels of aggression, the volleyball group showed markedly fewer hostile manifestations when resolving conflicts and expressing creative ideas (Trajković et al., 2020b). Harris and Reiter-Palmon (2015) noted that highly aggressive individuals are more inclined to generate creative solutions with manipulative or retaliatory intent in task contexts; however, in team-based settings, this tendency is constrained by cooperative norms and peer feedback (Harris and Reiter-Palmon, 2015). In line with the 6P model of malevolent creativity (Cropley et al., 2010), team sports participation—as a positive environmental factor—may optimize participants’ emotional states (“Person Feelings”) and personal characteristics (“Person Properties”) (Tamminen et al., 2016), and shape cooperative processes (“Process”), thereby reducing the likelihood that aggression is translated into malevolent creativity. Thus, we further hypothesize:

*Hypothesis 3b*: Team sports participation moderates the relationship between aggression and malevolent creativity among college students, such that the positive association between aggression and malevolent creativity is weaker at higher levels of team sports participation.

## 3 Methods

### 3.1 Participants and procedures

Shanghai is one of the most developed regions in China with respect to socio-economic development and higher education (Fu, 2025), and university students in this area are generally characterized by strong competitiveness and innovative capacity (Liang et al., 2016). Focusing on this population therefore helps enhance the representativeness and generalizability of the present findings.

In this study, college students were recruited from universities located in Yangpu University Town in Shanghai (including Fudan University, Tongji University, Shanghai University of Finance and Economics, Shanghai International Studies University, University of Shanghai for Science and Technology, and Shanghai University of Sport). With the support of the Student Affairs Offices at these universities, an online questionnaire was distributed via Wenjuanxing (Questionnaire Star), a widely used online survey platform in China. After reading and electronically signing the informed consent form, participants proceeded to complete the survey. The questionnaire was completed anonymously. The introduction clearly stated that there were no right or wrong answers and encouraged participants to respond honestly based on their actual situation. To reduce response bias, some items were reverse-worded; during data analysis, all items were recoded so that higher scores consistently indicated higher levels of the corresponding construct. Following Curran’s (2016) recommendations for questionnaire data quality control (Curran, 2016), we excluded 144 invalid responses with a completion time of less than 150 s or with identical responses to five or more consecutive items. The final valid sample consisted of 1,288 college students, yielding an effective response rate of 89.94%. Among them, 656 were male and 632 were female, indicating an approximately balanced gender ratio. The study protocol was approved by the Ethics Committee of Shanghai University (approval number: ECSHU: 2025–019).

### 3.2 Measures

#### 3.2.1 Social exclusion

Social exclusion was measured using the College Students’ Social Exclusion Questionnaire developed by Wu et al. (2013). This scale has been administered for over a decade in China and has demonstrated good reliability and validity. In the present study, several items were slightly revised to better reflect the developmental characteristics of contemporary college students and recent changes in interpersonal contexts on Chinese campuses. The revised scale consisted of 13 items (e.g., “On campus, I often notice classmates who, when feeling down, receive little comfort or consolation from others” and “I can sense that some classmates around me are always easily targeted for malicious teasing or pranks”).

All items were rated on a 5-point Likert scale (1 = “strongly disagree,” 5 = “strongly agree”), with higher total scores indicating higher perceived levels of social exclusion. In this study, the scale showed excellent internal consistency (Cronbach’s *α* = 0.95).

#### 3.2.2 Aggression

Aggression was assessed using the Aggression Questionnaire originally developed by Buss and Perry (1992) and later revised into Chinese by Liu et al. (2009). For the present study, we adapted the wording of some items to better match conflict situations commonly encountered by contemporary college students. The final version retained nine representative items (e.g., “I can sense that some classmates, when angry, often say hurtful things to others” and “I can sense that some classmates, when they are mad, tend to engage in impulsive violent behaviors such as kicking doors or throwing objects”).

Items were rated on a 5-point Likert scale (1 = “strongly disagree,” 5 = “strongly agree”), with higher scores reflecting higher levels of aggression. In the current sample, the scale demonstrated high internal consistency (Cronbach’s *α* = 0.94).

#### 3.2.3 Malevolent creativity

Malevolent creativity was measured using the Malevolent Creativity Behavior Scale developed by Hao et al. (2016) for Chinese college students. In light of recent changes in social environments and behavioral patterns, we updated several items to capture more contemporary forms of malevolent behavior. The final scale comprised nine items (e.g., “I have noticed classmates who are adept at using small but clever tricks to take revenge on people they dislike, such as anonymously spreading rumors on social media or using AI to generate fake chat logs or images”).

This scale assesses the tendency to engage in novel and covert behaviors in campus life to achieve negative or harmful goals. All items were rated on a 5-point Likert scale (1 = “strongly disagree,” 5 = “strongly agree”), with higher scores indicating higher levels of malevolent creativity. In the present study, the internal consistency of the scale was excellent (Cronbach’s *α* = 0.94).

#### 3.2.4 Team sports participation

Team sports participation was assessed based on the Group Environment Questionnaire (GEQ) developed by Carron et al. (1985). Drawing on this framework, and considering the specific context of Chinese university physical education courses, varsity team training, and sports club activities, we adapted and revalidated the instrument in collaboration with Chinese scholars. The revised scale included nine items that capture the socialization effects of team-sport contexts on students’ psychological and behavioral functioning (e.g., “Participation in team sports helps cultivate members’ sense of social belonging” and “Participation in team sports helps team members learn to regulate their emotions”).

Items were rated on a 5-point Likert scale (1 = “strongly disagree,” 5 = “strongly agree”). Higher total scores reflect a higher degree of team sports participation, indexed by stronger integration into team-based sport activities and higher levels of team identification. In this study, the scale showed excellent internal consistency (Cronbach’s *α* = 0.96).

## 4 Results

### 4.1 Test of common method bias

Because all data in the present study were collected via self-report questionnaires, common method bias may be a potential concern. To assess its severity, we conducted Harman’s single-factor test by performing an exploratory factor analysis on all items. The results showed that four factors had eigenvalues greater than 1, and the first unrotated factor accounted for 37.43% of the total variance, which is below the commonly used 40% threshold (Zhou and Long, 2004). This suggests that common method bias is unlikely to be a serious issue in the present data.

### 4.2 Descriptive statistics, correlations analysis and square root of the AVE

**Figure 1:**
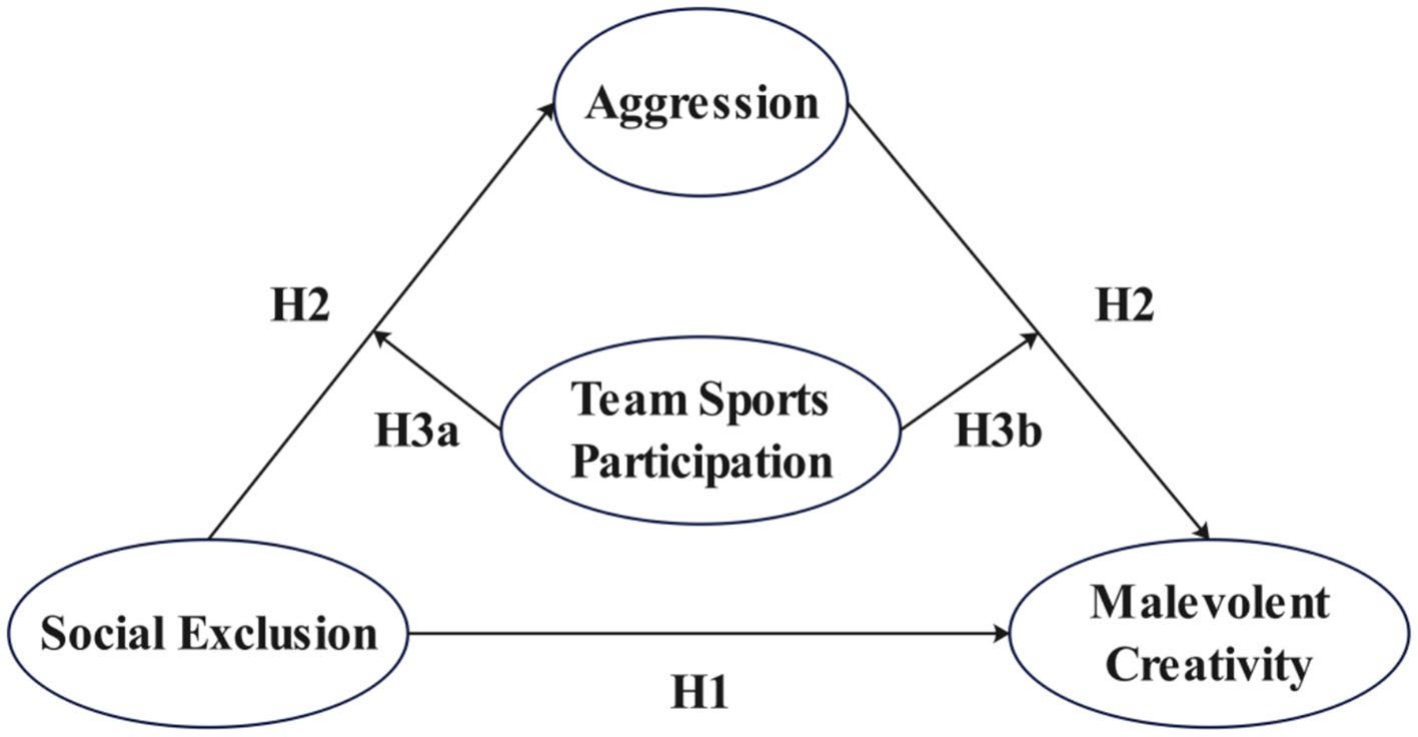
Hypothesized moderated mediation model of the effect of social exclusion on malevolent creativity among college students.

Descriptive statistics and bivariate correlations among the study variables are presented in Table 1. Social exclusion, aggression, and malevolent creativity were all positively and significantly correlated with one another. In contrast, team sports participation was significantly and negatively correlated with social exclusion, aggression, and malevolent creativity. The square root of the AVE for each construct was greater than the absolute value of its correlations with other constructs, and the AVE for each construct exceeded 0.50, indicating that the measurement model demonstrates good discriminant and convergent validity (Figure 1).

**Table 1:** Correlation analysis and the square root of AVE.

Variable	*M*	SD	1	2	3	4
1 Social exclusion	3.27	0.91	**0.7605**			
2 Aggression	3.37	0.93	0.43***	**0.7586**		
3 Team sports participation	2.75	1.01	−0.36***	−0.45***	**0.7885**	
4 Malevolent creativity	3.30	0.93	0.41***	0.44***	−0.47***	**0.7586**

***p < 0.001. Bolded values indicate the square roots of the average variance extracted (AVE).

### 4.3 Moderated mediation model

#### 4.3.1 Mediating effect of aggression

**Figure 2:**
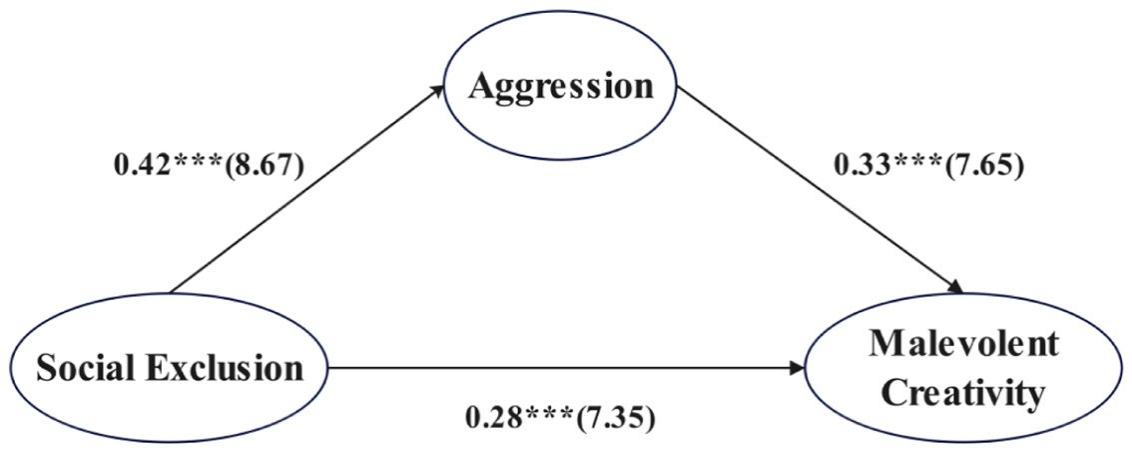
Mediation model of aggression in the relationship between social exclusion and malevolent creativity. ***p < 0.001.

Using Model 4 of Hayes (2012) PROCESS macro for SPSS (Hayes, 2012), we tested the mediating effect of aggression in the relationship between social exclusion and malevolent creativity among college students, controlling for gender, grade level, and age. The path coefficients among the variables are presented in Figure 2.

The results (Table 2) showed that social exclusion significantly predicted malevolent creativity among college students (total effect = 0.41, Boot 95% CI [0.36, 0.47]). When aggression was entered as a mediator, the direct effect of social exclusion on malevolent creativity remained significant (direct effect = 0.28, Boot 95% CI [0.22, 0.33]). In addition, the 95% bootstrap confidence intervals for both the direct effect and the indirect effect via aggression did not include zero, indicating that aggression played a partial mediating role in the relationship between social exclusion and malevolent creativity. The direct and indirect effects accounted for 68.29 and 31.71% of the total effect, respectively, suggesting that social exclusion not only directly predicts malevolent creativity but also exerts an additional effect through increased aggression. These findings support Hypothesis 2.

**Table 2:** Total, direct, and indirect effects of social exclusion on malevolent creativity via aggression.

Variable	Effect size	Boot SE	Boot LLCI	Boot ULCI	Effect proportion
Total effect	0.41	0.03	0.36	0.47	
Direct effect	0.28	0.03	0.22	0.33	68.29%
Indirect effect	0.13	0.01	0.11	0.16	31.71%

#### 4.3.2 Moderating effect of team sports participation

The results (Table 3) showed that, after including team sports participation in the mediation model, the interaction term between social exclusion and team sports participation (social exclusion × team sports participation) in the first stage significantly and negatively predicted aggression (*b* = −0.11, *t* = −4.70, *p* < 0.01). In the second stage of the model, the interaction term between aggression and team sports participation (aggression × team sports participation) also significantly and negatively predicted malevolent creativity (*b* = −0.11, *t* = −4.59, *p* < 0.01). These findings indicate that team sports participation moderates both the path from social exclusion to aggression and the path from aggression to malevolent creativity. Thus, Hypotheses 3a and 3b are supported.

**Table 3:** Tests of the moderated mediation model (unstandardized coefficients).

Variable	Aggression			Malevolent creativity		
	*b*	SE	*t*	*b*	SE	*t*
Social exclusion	0.59	0.07	8.67**	0.20	0.03	7.35**
Team sports participation	0.08	0.09	0.90	0.12	0.08	1.37
Aggression				0.51	0.07	7.65**
Social exclusion × Team sports participation	−0.11	0.02	−4.70**			
Aggression × Team sports participation				−0.11	0.24	−4.59**
*F*	96.90			93.01		
*R* ^2^	0.31			0.34		

**p < 0.01.

**Figure 3:**
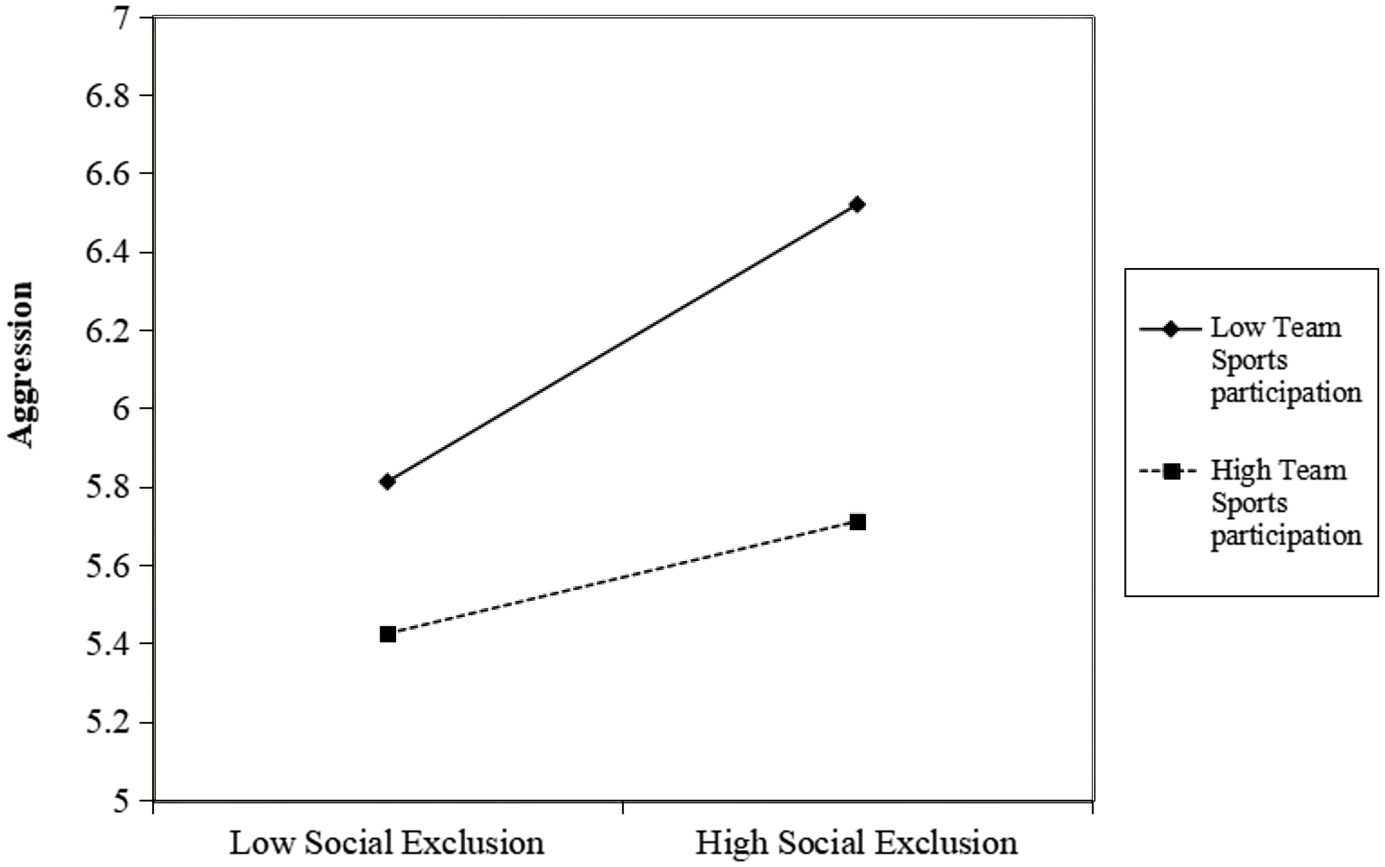
Moderating effect of team sports participation on the relationship between social exclusion and aggression.

To further clarify how team sports participation moderates the relationship between social exclusion and aggression, we divided participants into high and low team sports participation groups and conducted simple slope analyses. As shown in Figure 3, for college students with low levels of team sports participation, social exclusion significantly and positively predicted aggression (*β*_simple_ = 0.387, *t* = 11.842, *p* < 0.001). For those with high levels of team sports participation, the positive predictive effect of social exclusion on aggression remained significant but was markedly weaker (*β*_simple_ = 0.157, *t* = 4.047, *p* < 0.001). These results indicate a significant moderating effect of team sports participation on the link between social exclusion and aggression: the positive association between social exclusion and aggression is stronger among students with lower levels of team sports participation than among those with higher levels of participation.

**Figure 4:**
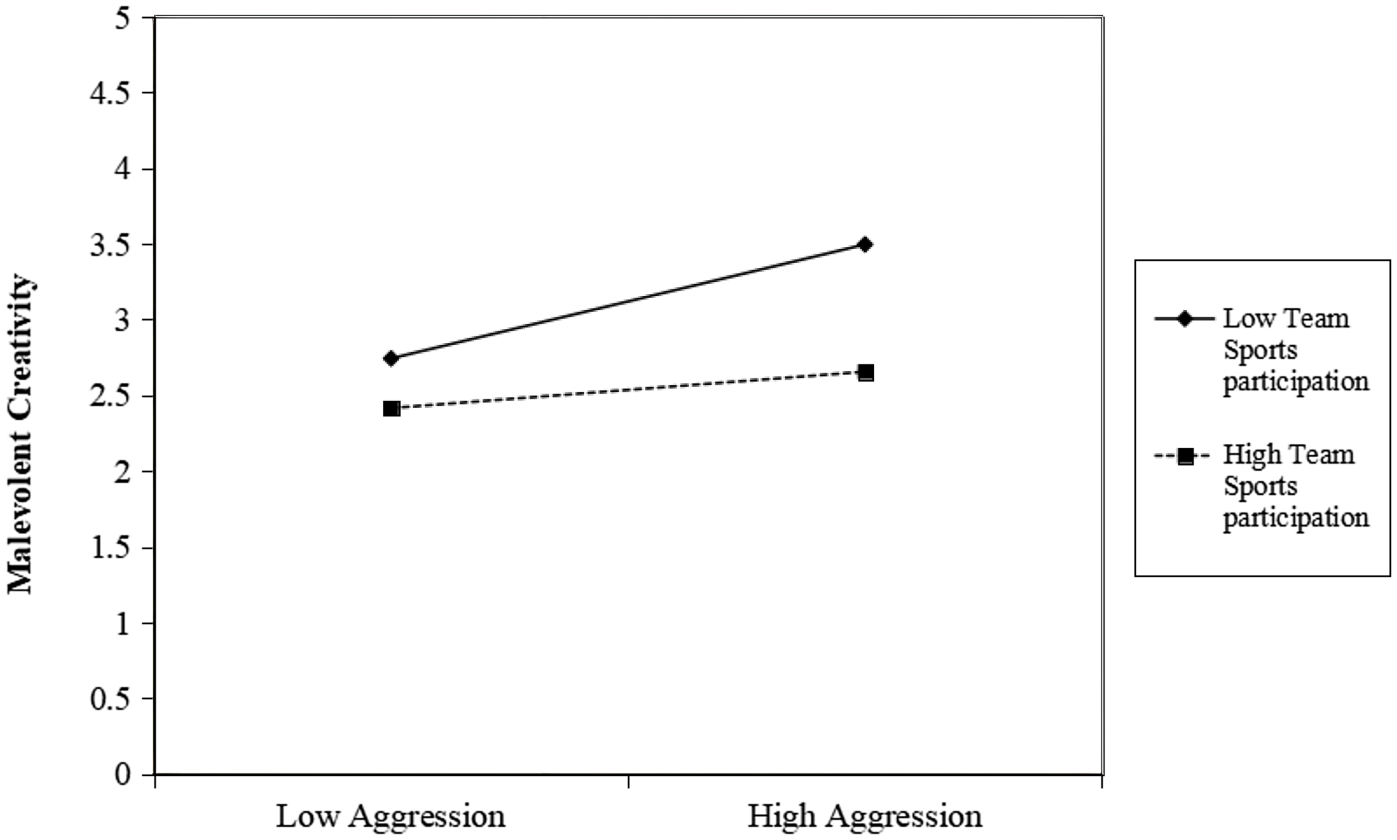
Moderating effect of team sports participation on the relationship between aggression and malevolent creativity.

To further examine how team sports participation moderates the relationship between aggression and malevolent creativity, participants were divided into high and low team sports participation groups, and simple slope analyses were conducted. As shown in Figure 4, among college students with low levels of team sports participation, aggression significantly and positively predicted malevolent creativity (*β*_simple_ = 0.403, *t* = 12.473, *p* < 0.001). Among those with high levels of team sports participation, the positive predictive effect of aggression on malevolent creativity remained significant but was substantially weaker (*β*_simple_ = 0.129, *t* = 3.228, *p* < 0.001). These findings indicate a significant moderating effect of team sports participation on the link between aggression and malevolent creativity: high levels of team sports participation can effectively buffer the adverse impact of aggression on malevolent creativity.

## 5 Discussion

### 5.1 The influence of social exclusion on the malevolent creativity among college students

The results of this study indicate that social exclusion positively predicts malevolent creativity among college students, thereby supporting Hypothesis 1. This finding is consistent with prior research (Li et al., 2024) and further corroborates that social exclusion, as a negative social context, may partially prompt individuals to unleash and deploy their creative potential in destructive ways (Perchtold-Stefan et al., 2024). On university campuses—where interpersonal interactions are frequent and social evaluation is highly salient—students are more likely to experience neglect, rejection, or exclusion from peers, organizations, or groups. Such experiences of social exclusion not only undermine students’ sense of belonging and social identification, but also may elicit a series of negative emotional reactions, including frustration, anger, and hostility (Lasko et al., 2022). Against this backdrop, individuals may be more inclined to channel their creative resources toward malevolent goals such as revenge, self-defense, or the restoration of power, thereby increasing the likelihood of malevolent creativity.

From the perspective of social information processing theory, the way individuals interpret and encode social cues directly shapes subsequent emotional responses and behavioral patterns (Gutworth, 2014). Within this framework, social exclusion continuously sends signals of “being treated unfairly,” leading excluded individuals to interpret others’ behaviors through a hostile lens and to reduce their level of trust in the social environment (Thériault et al., 2023). Under the influence of such hostile attribution biases, and in the presence of negative emotions such as frustration and anger, excluded individuals are more likely to invest their creative resources in designing malevolent strategies against others or groups, using harm and destruction as means of psychological compensation and emotional release (Bernstein et al., 2018). Moreover, contemporary college students are in a critical period of socialization and identity construction: they possess high cognitive flexibility and growing independence (Sawyer et al., 2012), yet have relatively limited social experience, and their emotion regulation and moral judgment capacities are still developing (Heynen et al., 2025). In this developmental context, when students encounter exclusion in campus settings but lack effective emotion regulation strategies and social support, they are more prone to apply creative thinking to hostile expression and the generation of harmful strategies, thereby elevating the risk of malevolent creativity.

This risk is particularly salient in the current era of rapid technological advancement. With the proliferation of digital technologies and virtual platforms, the anonymity and technical affordances of cyberspace provide greater concealment and operability for harmful behaviors, rendering manifestations of malevolent creativity more diverse and more destructive (d'Amato A. L. et al., 2025; Ge, 2022). Although Hypothesis 1 aligns with earlier empirical findings, it must be interpreted in light of China’s rapid social transformation: under the dual pressures of a fast-paced lifestyle and accelerating technological development, behaviors related to malevolent creativity appear to be evolving at an increasingly rapid rate (Bajwa, 2022; Cai, 2024). In particular, as artificial intelligence technologies become widely integrated into higher education, the forms and pathways of malevolent creativity are becoming more diversified, refined, and covert (d'Amato A. et al., 2025; Theobald et al., 2024), and the resulting harm is more difficult to anticipate and prevent. These emerging patterns of malevolent creativity—jointly driven by structural social change and technological power—have already exceeded the contextual boundaries depicted in traditional research and therefore merit sustained attention and serious consideration in future theoretical work and in university education and management practice.

### 5.2 The mediating role of aggression

The present study found that aggression plays a significant mediating role in the mechanism through which social exclusion influences malevolent creativity among college students, thereby supporting Hypothesis 2. This result is consistent with previous empirical findings (Baas et al., 2019; Harris and Reiter-Palmon, 2015; Lee and Dow, 2011) and accords with the theoretical expectations of the General Aggression Model. Unlike much of the existing work that has focused on workplace settings and emphasized the mediating role of aggression under conditions of conflicts of interest and promotion pressure (Aquino and Thau, 2009; Neuman, 2004), the university campus represents a markedly different social context in terms of relational structures and types of conflict. College students’ interests and aspirations tend to be more diverse and exploratory, and their interpersonal tensions and conflicts are more likely to manifest as discrepancies between ideals and reality (Mack, 2018), confusion and fragmentation in self-identity (Osteen, 2011), and frictions arising from differences in daily habits (Anju et al., 2021). These factors can also accumulate into social exclusion experiences that foster and intensify aggression, but their connotations differ from the more instrumental, utility-driven dynamics typically observed in organizational settings. In recent years, a number of incidents reported in Chinese universities—where social exclusion has triggered malevolent creativity that was successfully enacted and led to adverse outcomes—have, to some extent, provided real-world evidence for the existence of this mediating pathway.

More specifically, social exclusion, as a negative social stimulus, undermines individuals’ sense of belonging and social connectedness (Baumeister et al., 2007), thereby eliciting emotional reactions such as anger and hostility and elevating levels of aggression (Chester and DeWall, 2017; DeWall et al., 2009; Hames et al., 2018). Arnett’s theory of emerging adulthood suggests that ages 18–25 constitute a distinct developmental period between adolescence and adulthood, characterized by intensified identity exploration, instability in life circumstances, and a high degree of self-focus (Arnett, 2000). In this stage, aggression is not only expressed through direct venting and retaliation, but can also be understood as a distorted assertion of autonomy or a resistant response to a perceived “devalued identity.” Once activated, aggression may profoundly shape individuals’ cognitive processing, orienting them toward harmful goals as the target of innovation and prompting them to invest creative resources in the design of destructive strategies, ultimately fostering the emergence of malevolent creativity. This finding further supports the dual-pathway perspective on creativity, which posits that both positive and negative emotions can promote creative performance via different mechanisms (Nijstad et al., 2010).

### 5.3 The moderating role of team sports participation

The present study also verified the moderating effect of team sports participation on the path from social exclusion to aggression. The interaction between social exclusion and team sports participation significantly and negatively predicted aggression among college students (*t* = −4.70, *p* < 0.01). In other words, higher levels of team sports participation attenuated the positive association between social exclusion and aggression, indicating that team sports participation plays an important buffering role in this pathway. Notably, although previous studies have discussed the potential of team sports participation to enhance social acceptance (Andersen et al., 2019; Wann, 2006) and reduce the negative effects of social exclusion (Oettle and Greiner, 2025), few have directly tested its moderating mechanism in the process by which social exclusion contributes to aggression. The present findings help fill this gap. Some existing research has examined the social acceptance benefits associated with team sports participation at the individual level. For example, Boone and Leadbeater (2006) suggested that team sports participation may increase college students’ perceived social acceptance and reduce social pain and other negative consequences of exclusion (Boone and Leadbeater, 2006), but they did not provide direct statistical evidence for a moderating effect of team sports participation. Other scholars have compared team and individual sports in more fine-grained ways. Nielsen et al. (2014) found that making new friends was a salient benefit of participating in team games such as football, whereas male participants in individual activities like spinning did not report this advantage, and team sports appeared more intrinsically motivating than individual fitness activities (Nielsen et al., 2014). Chacón-Cuberos et al. (2020) further showed that, following experiences of rejection, individuals who regularly participated in team sports reported lower levels of negative affect and stronger emotion regulation abilities (Chacón-Cuberos et al., 2020).

However, these studies have primarily focused on how team sports participation promotes prosocial functioning, while paying relatively little attention to its role in suppressing aggression and, through reduced aggression, preventing subsequent socially destructive behaviors. This gap is particularly concerning in the current era, in which artificial intelligence is becoming widespread and information is increasingly easy to access and manipulate. Under such conditions, high-ability college students are especially well positioned to exploit complex information technologies to engage in sophisticated forms of social harm, highlighting the urgency of understanding and leveraging the protective role of team sports participation in the social exclusion–aggression–malevolent creativity pathway.

The present study also verified the moderating effect of team sports participation on the path from aggression to malevolent creativity among college students. The interaction term between aggression and team sports participation significantly and negatively predicted malevolent creativity (*t* = −4.59, *p* < 0.01). This finding indicates that the higher the level of team sports participation, the weaker the facilitative effect of aggression on malevolent creativity. In other words, team sports not only buffer the influence of external stressors on aggression, but also interfere with the further transformation of aggressive tendencies into malevolent creative behavior. Existing research provides important theoretical support for this conclusion. When individuals’ aggressive impulses can be appropriately expressed and channeled, the underlying aggressive energy can be released in a relatively safe manner (Liu, 2022), and participation in physical exercise is widely recognized as a positive and healthy way to regulate and vent such impulses (Wang, 2017). According to Cropley et al.’s (2010) 6P model of malevolent creativity, the emergence of malevolent creativity involves a psychological “output” process in which individuals, within specific social environments, accumulate negative emotions over time and gradually form ideas with harmful intent; once this output has been sufficiently reinforced cognitively and emotionally, it is more likely to be enacted through self-justification and influence on others. Team sport participation can reshape the expression of malevolent creativity not merely through the exercise per se, but because it simultaneously supplies affective and social protective resources. On the one hand, engaging in sport is associated with higher positive affect and subjective well-being and with lower psychological distress, which can dampen the escalation of exclusion-related negative affect and the subsequent arousal of aggression (Liu et al., 2023). On the other hand, the shared goals and high-density interactions inherent in team sports are more likely to foster social connection and perceived support (Scott et al., 2024), while structured training routines and rule- and tactic-based demands are linked to stronger executive functioning, especially inhibitory control and impulse regulation, making it harder for aggressive impulses to crystallize into novel, harmful ideas (De Waelle et al., 2021; Fan et al., 2021). In doing so, team sports participation intervenes in the formation of malevolent creativity at both the environmental and individual levels, functioning as a kind of “safety valve” for emotion regulation and behavioral control.

Taken together, team sports participation not only represents a positive and healthy lifestyle that helps buffer students’ psychological risks when facing adverse environments, but also serves, to some extent, to weaken the driving role of aggression in the development of malevolent creativity, thereby interrupting the pathway through which negative emotions and maladaptive traits are transformed into destructive creative behaviors.

## 6 Conclusion

(1) The findings show that social exclusion significantly and positively predicts malevolent creativity among college students. Aggression serves as a mediator in this relationship, indicating that social exclusion can indirectly heighten students’ malevolent creative tendencies by increasing their level of aggression.

(2) Higher levels of team sports participation attenuate the impact of social exclusion on aggression and, likewise, weaken the effect of aggression on malevolent creativity. This suggests that active engagement in team sports may help reduce malevolent creativity among college students by buffering the adverse psychological and behavioral consequences of social exclusion and aggression.

## 7 Implications and limitations

The present study demonstrates that team sports participation, as a socialized and structured positive activity, can substantially weaken aggressive responses triggered by social exclusion and inhibit the further translation of aggression into malevolent creativity. This extends the application boundary of physical activity within the field of psychological and behavioral intervention. On this basis, universities may systematically integrate sport-related resources with mental health education by encouraging students’ sustained engagement in team sports. Such engagement can enhance students’ sense of belonging and emotion regulation abilities, while simultaneously providing positive interpersonal feedback and clear behavioral norms. In turn, this may help cultivate a campus climate that channels creativity toward prosocial ends and offers a novel practical pathway for preventing and intervening in malevolent creative behaviors.

Nonetheless, several limitations of this study should be noted. First, the cross-sectional survey design reflects a static snapshot and, although it reveals the relational pattern among social exclusion, aggression, malevolent creativity, and team sports participation, causal inferences remain tentative and need to be further tested through longitudinal or experimental studies. Second, both malevolent creativity and aggression were primarily assessed using self-report scales, which may be subject to social desirability effects and self-presentation biases. Future research could incorporate behavioral tasks, physiological indicators, or informant reports to enhance the reliability and validity of the findings and to further enrich our understanding of the mechanisms underlying malevolent creativity among college students as well as potential avenues for effective intervention. Finally, although this study conceptualized social exclusion as an antecedent of malevolent creativity, we did not control for other variables that may be associated with social exclusion and could jointly influence malevolent creativity. This may have led to an overemphasis on the unique role of social exclusion. Future research should incorporate additional relevant covariates and conditional factors to more rigorously evaluate the relationship between social exclusion and malevolent creativity.

## Data Availability

The raw data supporting the conclusions of this article will be made available by the authors, without undue reservation.
